# Association between sarcopenia and outcomes of surgically treated oral squamous cell carcinoma: a systematic review and meta‐analysis

**DOI:** 10.3389/fonc.2024.1445956

**Published:** 2024-11-01

**Authors:** Kai Luo, Kaiming Chen, Yu Li, Yang Ji

**Affiliations:** ^1^ Department of Anesthesiology, West China Hospital of Stomatology, Sichuan University, Chengdu, China; ^2^ Department of Anesthesiology, West China Hospital, Sichuan University, Chengdu, China

**Keywords:** oral squamous cell carcinoma, curative resection, sarcopenia, overall survival, outcomes

## Abstract

**Background:**

Sarcopenia is a significant predictor of perioperative adverse outcomes for a variety of malignancies and has significant negative effects on surgical and oncology outcomes. The development of sarcopenia is mainly attributed to aging, inactivity, poor nutrition, and decreased testosterone levels, which suggest a poor prognosis after surgery. Therefore, the primary objective of this systematic review and meta-analysis was to determine the effect of sarcopenia on postoperative survival in patients with oral squamous cell carcinoma.

**Methods:**

We systematically searched databases including PubMed, Embase, Cochrane Library, Medline and Web of Science from inception to 12 July 2023, to determine the prognostic value of sarcopenia in oral squamous cell carcinoma. The primary outcome was three-year survival, and secondary outcomes were one-year survival, five-year survival, infection and pneumonia within 30 days postoperatively. Original studies comparing postoperative outcomes in patients with sarcopenia and non-sarcopenia for oral squamous cell carcinoma curative therapy were met the eligibility criteria. We used Endnote X9 for the screening process and used RevMan 5.4.1 for our meta-analysis, all results in this study were performed using a random-effects model. QUIPS (Quality in Prognosis Studies) tools and GRADE (Grading of Recommendations, Assessment, Development and Evaluations) were used for risk of bias and quality of evidence assessment.

**Result:**

Ten original studies with 50611 patients met the inclusion criteria. Meta-analysis showed that patients with sarcopenia reduced three-year OS after surgery (OR = 0.73, 95% CI = 0.66-0.81, P < 0.00001). The one-year OS (OR = 0.71, 95% CI = 0.67-0.75, P < 0.00001) and five-year OS (OR = 0.60, 95% CI = 0.45-0.79, P = 0.0003) decreased significantly. Patients with sarcopenia had significantly increased 30-day postoperative mortality and an also increased risk of pneumonia (OR = 1.36, 95% CI = 1.24-1.49, P < 0.00001) and surgical site infection (OR = 2.49, 95% CI = 1.06-5.84, P = 0.04).

**Conclusion:**

Sarcopenia is associated with reduced survival in patients after curative resection. Meanwhile, 30-day mortality, postoperative pneumonia and surgical site infection were significantly higher than those in nonsarcopenic patients. Sarcopenia as an extremely important factor of postoperative adverse outcomes in OSCC patients need special attention.

**Systematic review registration:**

https://www.crd.york.ac.uk/PROSPERO/, identifier CRD42023444424.

## Introduction

Oral squamous cell carcinoma (OSCC) accounts for 90% of all oral carcinoma cases (1, 2). Despite advances in cancer diagnosis and surgical treatment, the five-year overall survival rate for OSCC remains below 50%, making it the lowest among oral malignancies. Unfortunately, its prognosis has shown little improvement over the past three decades (3, 4). The rapid progression of the disease and gaps in population screening programs for oral cancer mean that nearly 70% of oral cancer patients receive a diagnosis at an advanced stage, and often accompanied by weakness, malnutrition, and sarcopenia (5).

Sarcopenia, characterized by reduced physical activity, malnutrition, and systemic inflammatory responses, independently correlates with several adverse outcomes in head and neck cancer, including complications within 30 days after surgery and dose-restricted chemotherapy toxicity (6). Such consequences may contribute to the overall increase in mortality and reduced disease-free survival among patients with malignant tumors (7–10).

Numerous techniques have been proposed to evaluate sarcopenia. According to the European Working Group on Sarcopenia in Older People (EWGSOP) and the revised diagnostic consensus and management guidelines EWGSOP2, the cross-sectional area of the lumbar muscle, as measured through abdominal computed tomography (CT) scans, is an internationally recognized simple and reliable indicator for the diagnostic criteria of sarcopenia (11). In clinical practice, the imaging tests required for diseases bring convenience to the diagnosis of sarcopenia, and the psoas cross-sectional area determined by the computerized tomography (CT) scan imaging parameters from L3/L4 has become the most practical imaging measurement (12, 13). Additionally, bioelectrical impedance analysis (BIA) is widely utilized for disease screening due to its affordability and practicality (14).

The causes of sarcopenia include aging, endocrine and metabolic disorders and malnutrition (15). Patients affected by head and neck cancer are commonly associated with low muscle mass due to symptoms related to the location of these tumors, such as anorexia, fatigue and dysgeusia, have a higher risk of severe malnutrition with subsequently associated sarcopenia (16, 17). Sarcopenia, which hinders rapid perioperative recovery, not only impairs activities of daily living (18) and leads to reduced quality of life (19) but also increases the risk of perioperative respiratory disease (20), cognitive dysfunction (21) and reduced motor capacity (22). Some studies have found that sarcopenia was associated with reduced postoperative survival compared with patients without sarcopenia in surgical intervention and combined treatment of multiple malignancies (23–27). However, there has been no definitive conclusion on the impact of overall survival and postoperative adverse events on OSCC patients with sarcopenia. To address this gap, we conducted a meta-analysis of overall survival after radical OSCC surgery to evaluate the association between sarcopenia and overall survival in this context.

## Materials and methods

### Literature retrieval

We prepared this systematic review and meta-analysis in accordance with the latest Preferred Reporting Items for Systematic Reviews and Meta‐Analyses (PRISMA) requirements and guidelines (28). Two authors, LK and CMK, systematically searched the PubMed, Embase, Cochrane Library, Medline, and Web of Science databases to retrieve original studies of all patients with sarcopenia who underwent radical OSCC surgery from inception to July 12, 2023. Prior to retrieval, the agreement was registered with PROSPERO (registration number: CRD42023444424). We meticulously screened the original studies based on the inclusion and exclusion criteria, and any discrepancies between the two authors were resolved by a third author (JY). Additionally, we thoroughly searched the reference lists of all identified publications for additional relevant studies. The original research was limited to English literature. and the searches were rerun prior to the final analyses. A comprehensive search strategy is provided in the appendix.

### Eligibility criteria

The inclusion and exclusion criteria were established through negotiation by three investigators (LK, CMK, and JY) prior to registration. The studies that met the eligibility criteria were retained.

1. The original research was limited to English literature.
2. We focused on radical surgical treatment for OSCC.
3. Exposure factors were defined as sarcopenia, and nonexposure factors or controls were defined as nonsarcopenia.
4. Outcomes included at least one of three-year OS, one-year OS, five-year OS, 30-day mortality, surgical site infection, and pneumonia after surgery.

Articles that met the following criteria were excluded:

1. Animalistic study.
2. The study types were case reports, conference abstracts, reviews, letters or comments, and original records with inaccessible data were excluded.

### Data extraction

Two researchers independently extracted data from the included studies after completing qualification checks. Data extraction was performed according to a pre-specified protocol, which included details such as author, publication year, study design, age, sex, exposure and control sample size, diagnostic criteria, and outcomes. The primary outcome assessed was three-year overall survival (OS), while secondary outcomes included one-year OS, five-year OS, 30-day mortality, surgical site infection, and pneumonia after surgery. In cases of disagreement between the two researchers (LK and CMK) during manuscript screening and data extraction, consultation with the third author (JY) was used to resolve any discrepancies.

### Methodological quality

Similarly, two investigators (LK and CMK) independently assessed the quality of the included original studies and evaluated the risk of bias. We employed the Quality in Prognosis Studies (QUIPS) (29, 30) framework to assess the risk of bias in the included studies. QUIPS is based on recommendations from a comprehensive review of the quality of prognostic systematic reviews. When evaluating the validity and bias of prognostic factor studies, six critical areas are considered: study participation, study attrition, prognostic factor measurement, confounding measurement, outcome measurement, and analysis and reporting. For each of these six domains, responses to the prompting items were combined to inform judgments about the risk of bias, and each potential bias domain was rated as having a high, moderate, or low risk of bias.

Ultimately, we assessed the manuscript quality of evidence for three-year OS, one-year OS, five-year OS, 30-day postoperative mortality, infection and pneumonia developed within 30 days postoperatively. These outcomes were categorized into high, moderate, low, and very low levels based on the rating of the Grades of Recommendation, Assessment, Development, and Evaluation (GRADE) (31) approach and the grading of the quality and strength of evidence in the clinical practice guidelines for interventions. In accordance with the GRADE approach, factors such as research methodology, consistency and accuracy of results, and directness of evidence were all carefully considered. All disagreements were settled by consensus.

### Data analysis

We performed the meta-analysis using RevMan 5.4.1, and a random effects model was used to evaluate the difference in outcomes between sarcopenia and nonsarcopenia. A two-tailed P value of less than 0.05 was defined as statistically significant. Since all the specified outcomes were binary variables, odds ratios (ORs) were calculated. Heterogeneity among manuscripts was assessed using a combination of clinical and methodological methods, with I^2^ values indicating levels of heterogeneity: < 25% for low, 25%-50% for medium, and > 50% for high heterogeneity. We performed subgroup analysis based on the assessment of muscle mass, considering the varying diagnosis of sarcopenia. To further evaluate the robustness of our final results, we employed the leave-one-out method for sensitivity analysis, identifying and analyzing specific sources of heterogeneity. In cases where meta-analysis was not feasible due to data source limitations, we conducted systematic reviews by describing study outcomes.

## Results

### Study selection

**Figure 1:**
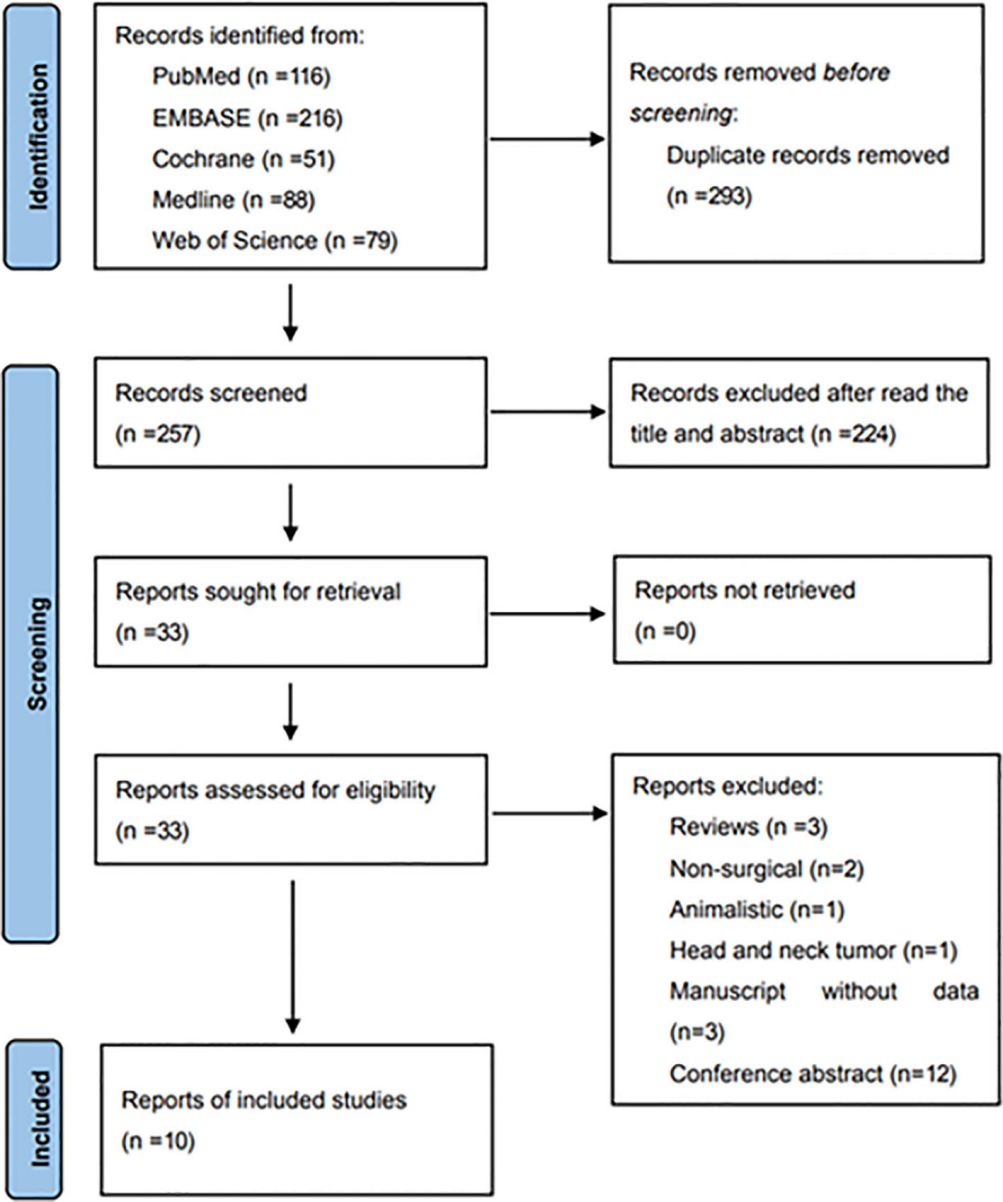
Flow diagram of study selection for inclusion in this systematic review and meta-analysis.

A systematic search yielded 550 original articles, and 257 records were identified after removing duplicate studies. Thirty-three studies were retained after title and abstract screening, followed by a detailed review the full text in accordance with the prespecified inclusion and exclusion criteria. Ultimately, ten eligible manuscripts, involving a total of 50,611 patients, were included in the systematic review and meta-analysis. **Figure 1** depicts the flow chart of literature screening.

### Characteristics of enrolled studies

Ten retrospective original studies, involving a total of 50,611 patients diagnosed with oral squamous cell carcinoma (OSCC) and who underwent radical surgery, were ultimately determined to meet the inclusion and exclusion criteria (5, 10, 32–39). Sarcopenia was reported in 17264 patients (34.1%). The L3/4 level skeletal muscle mass (SMM) or skeletal muscle index (SMI) estimated by CT scan was used to diagnose sarcopenia in seven records (5, 32–34, 36–38), and BIA was employed to assess body composition for the remaining studies (10, 35, 39). The definition of sarcopenia was performed according to the criteria of EWGSOP and EWGSOP2. All the included studies were published within the past three years, and sarcopenia emerged as a new prognostic factor in the field of clinical research. Online **Supplementary Data Sheet 1** provides the baseline patient characteristics and the details of body composition methodology and outcomes in the included studies.

### Risk of bias

Quality in Prognosis Studies (QUIPS) with detailed entry and applied to various types of study design, addresses six critical areas (study participation, study attrition, prognostic factor measurement, confounding measurement, outcome measurement, analysis and reporting), was designed for systematic reviews of prognostic factor studies. Online **Supplementary Data Sheet 2** (**Supplementary Table 3**) summarizes the risk of bias for the ten included studies. The primary source of bias is confounding measurement, followed by study attrition. Notably, this methodological weakness is most common in retrospective designs.

### Quality assessment

**Figure 2:**
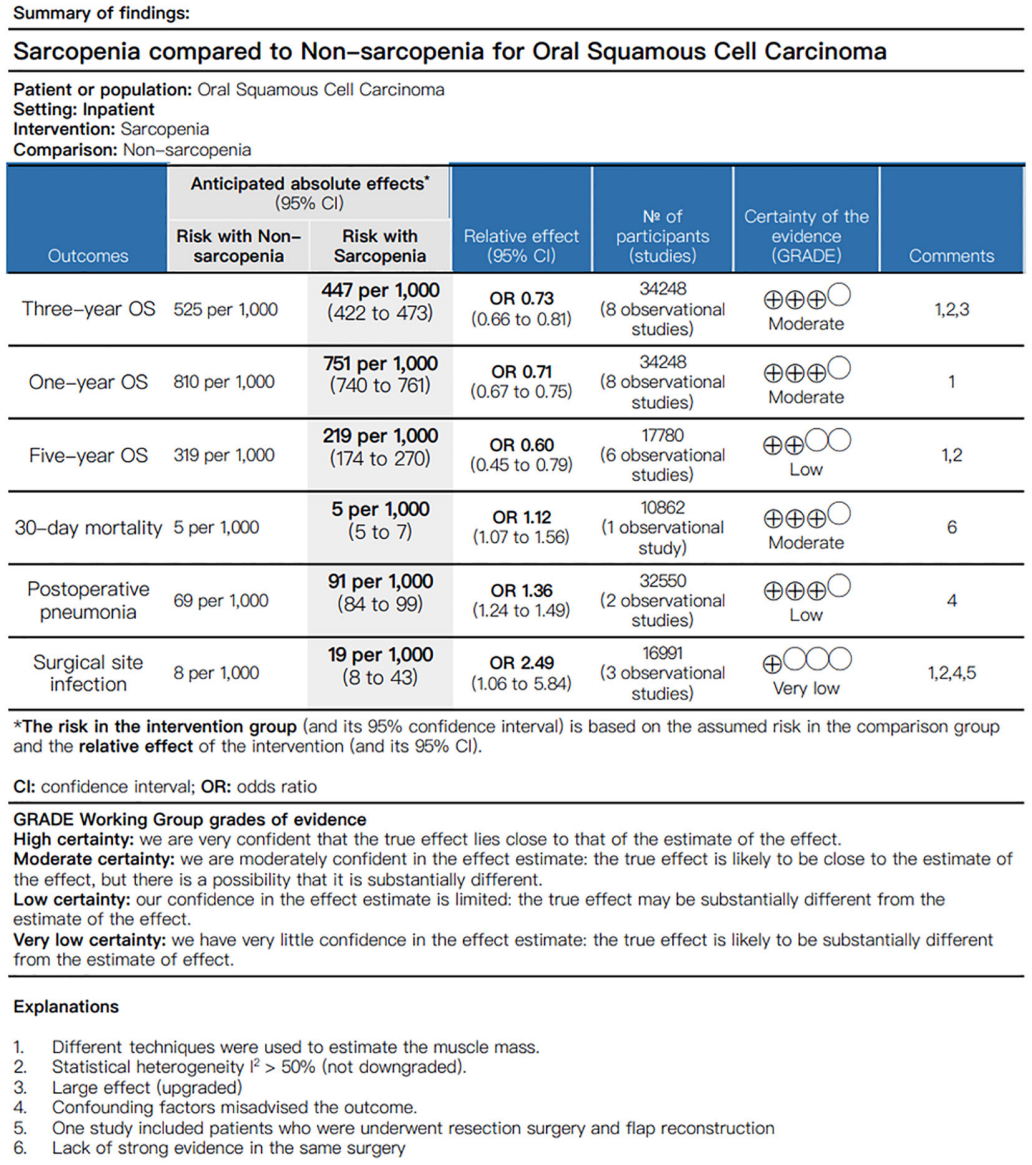
Certainty of the evidence and summary of findings.

We performed the quality of evidence and the strength of recommendations in accordance with GRADE guidelines and utilized GRADEpro to generate the Summary of Findings (SoF). The GRADE approach provides guidance to grading the quality of underlying evidence and the strength of recommendations in health care, and the system classifies quality of evidence as high, moderate, low, or very low according to factors that include the study methodology, consistency and precision of the results, and directness of the evidence. The GRADE grading system indicated that the quality of evidence for three-year OS, one-year OS and 30-day mortality was moderate. However, the quality of evidence for five-year OS and postoperative pneumonia was low, and the quality of evidence for surgical site infection was rated as very low (**Figure 2**).

### Primary outcome

#### Three-year OS

**Figure 3:**
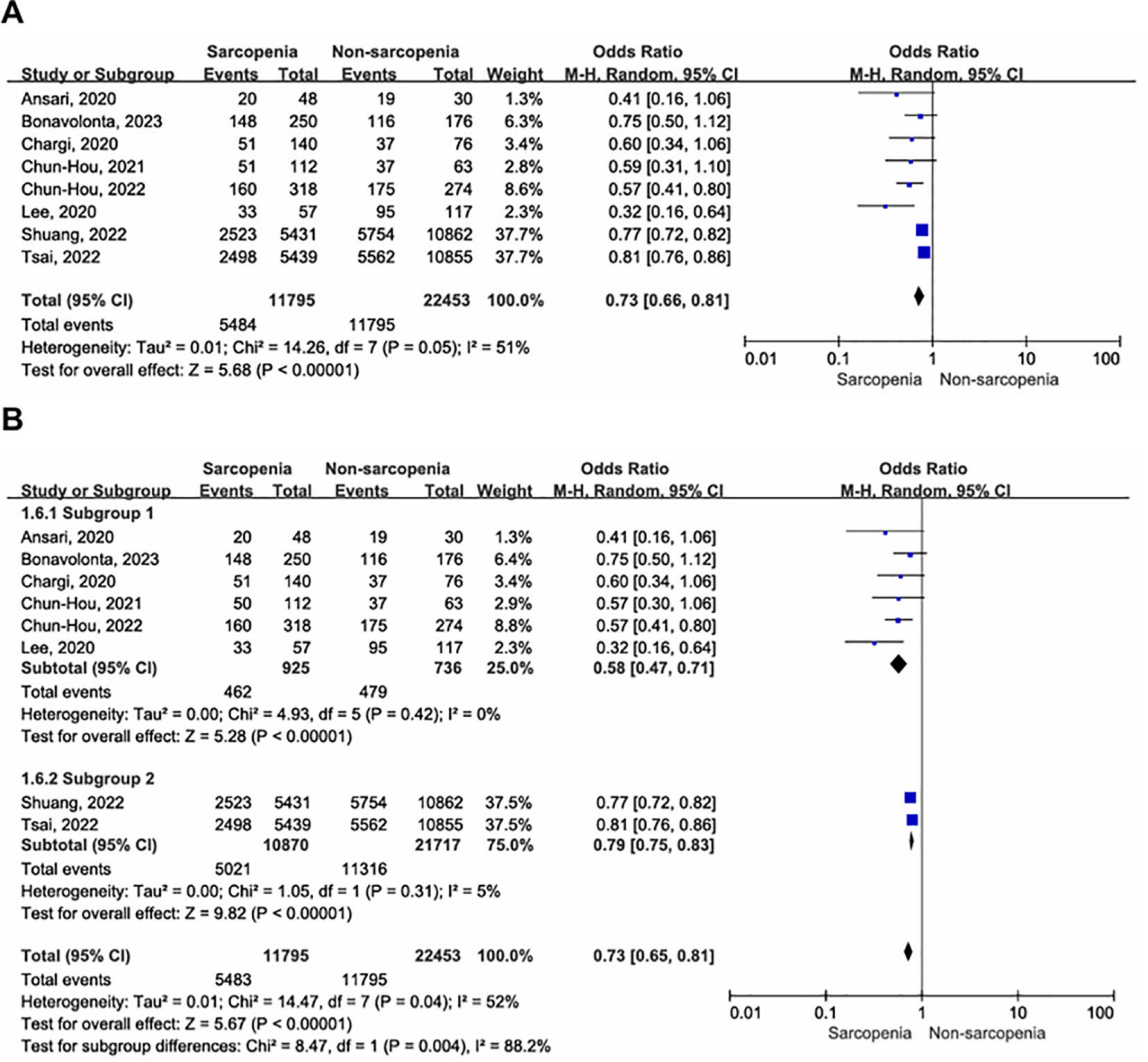
Forest plot of the primary outcome. **(A)** Forest plot of three-year OS; **(B)** Forest plot of three-year OS according to inconsistent measurement for sarcopenia, subgroup 1: sarcopenia defined by SMM at L3, subgroup 2: sarcopenia defined by BIA.

A total of eight articles reported three-year OS in patients with OCSCC undergoing curative surgery; Among these, 11,795 patients were diagnosed with sarcopenia, while 22,453 were diagnosed with nonsarcopenia (5, 10, 32–34, 36, 37, 39). Two enrolled studies reported that sarcopenia was diagnosed using BIA (10, 39), while the remaining studies used SMM at the level of C3, which was then converted to SMM at the level of L3. Compared with patients without sarcopenia, those with sarcopenia had a worse three-year OS (**Figure 3A**; OR = 0.73, 95% CI = 0.66-0.81, P < 0.00001). Significant heterogeneity was present in the included literature (I^2^ = 51%, P = 0.05).

Given the absence of a gold standard for the diagnosis of sarcopenia, we conducted a subgroup analysis to assess the impact of different sarcopenia diagnostic criteria on outcomes. The results revealed no evidence of heterogeneity within subgroups (**Figure 3B**). Sarcopenia, whether defined by SMM at the L3 level (OR = 0.58, 95% CI = 0.47-0.71, P < 0.00001; I^2^ = 0%, P = 0.42) or BIA (OR = 0.79, 95% CI = 0.75-0.83, P < 0.00001; I^2^ = 5%, P = 0.31), was associated with worse three-year postoperative survival in patients with OSCC, with significantly reduced heterogeneity between studies.

### Secondary outcomes

#### One-year OS

**Figure 4:**
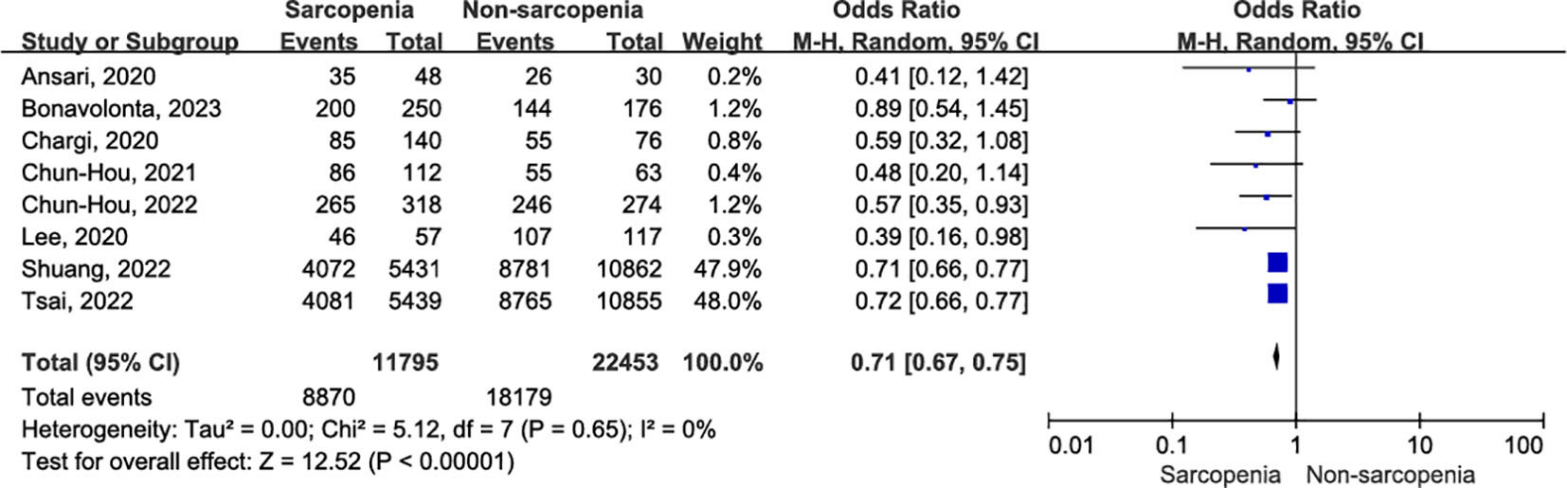
Forest plot of one-year OS.

Eight studies, involving 34,248 patients, provided evidence on the association between one-year OS and sarcopenia (5, 10, 32–34, 36, 37, 39). There was no evidence of heterogeneity (I^2^ = 0%, P = 0.65) among the recruited studies. Using a random effects model, we found that patients diagnosed with sarcopenia exhibited a significant reduction in one-year OS compared to those without sarcopenia (**Figure 4**; OR = 0.71, 95% CI = 0.67-0.75, P < 0.00001).

**Figure 5:**
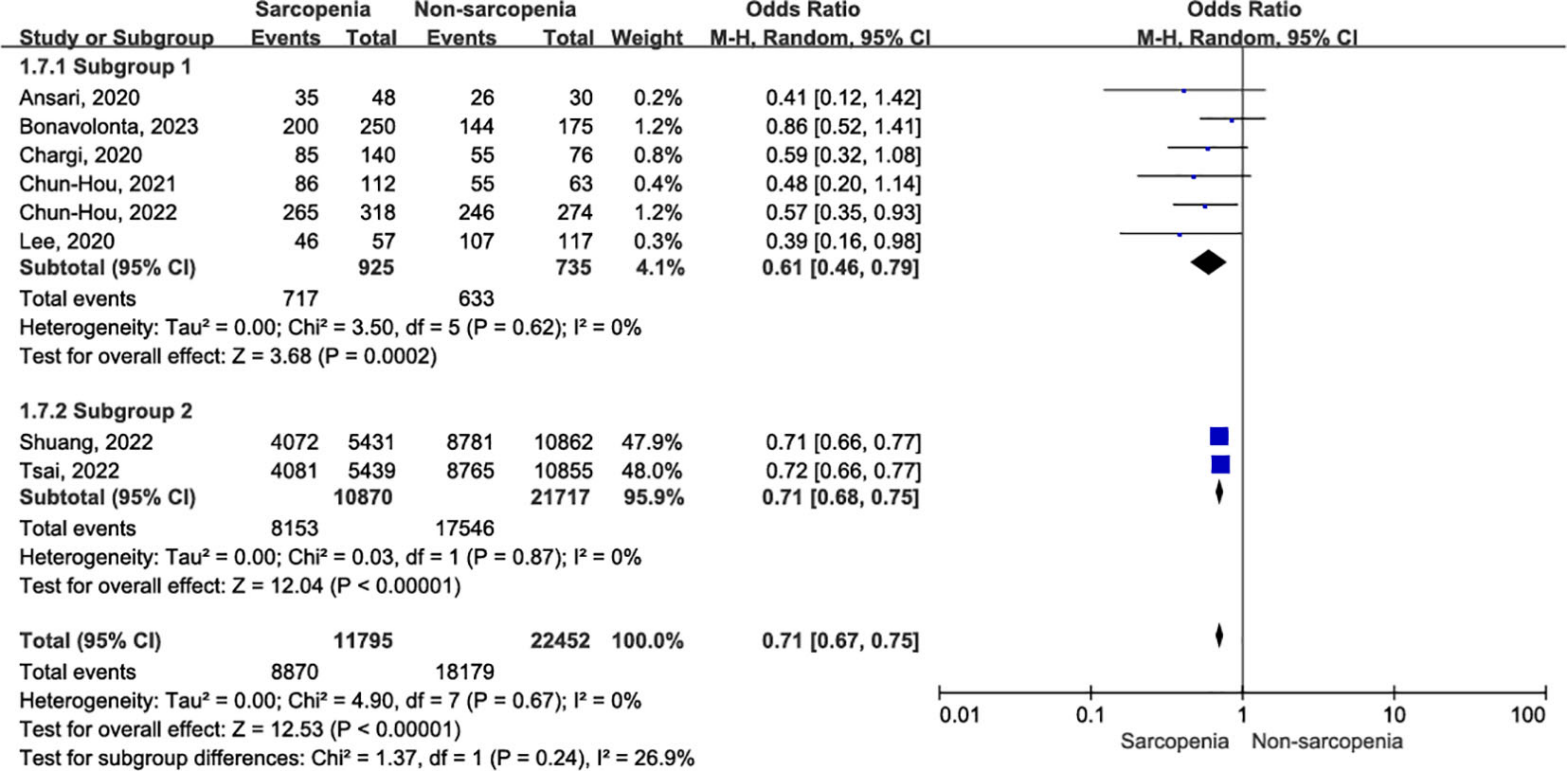
The forest plot of one-year OS according to inconsistent measurement for sarcopenia.

Similarly, in studies reporting one-year OS after OSCC surgery, the assessment of sarcopenia included both BIA (10, 39) and SMM. **Figure 5** illustrates the one-year OS for subgroup analysis based on sarcopenia assessment. Specifically, sarcopenia defined by SMM at L3 was significantly associated with decreased one-year OS in patients with OSCC (OR = 0.61, 95% CI = 0.46-0.79, P = 0.0002; I^2^ = 0%, P = 0.62), and this association was uniformly observed when sarcopenia was assessed using BIA (OR = 0.71, 95% CI = 0.68-0.75, P < 0.00001; I^2^ = 0%, P = 0.87). There was no evidence of heterogeneity within subgroups (I^2^ = 0%, P = 0.67).

#### Five-year OS

**Figure 6:**
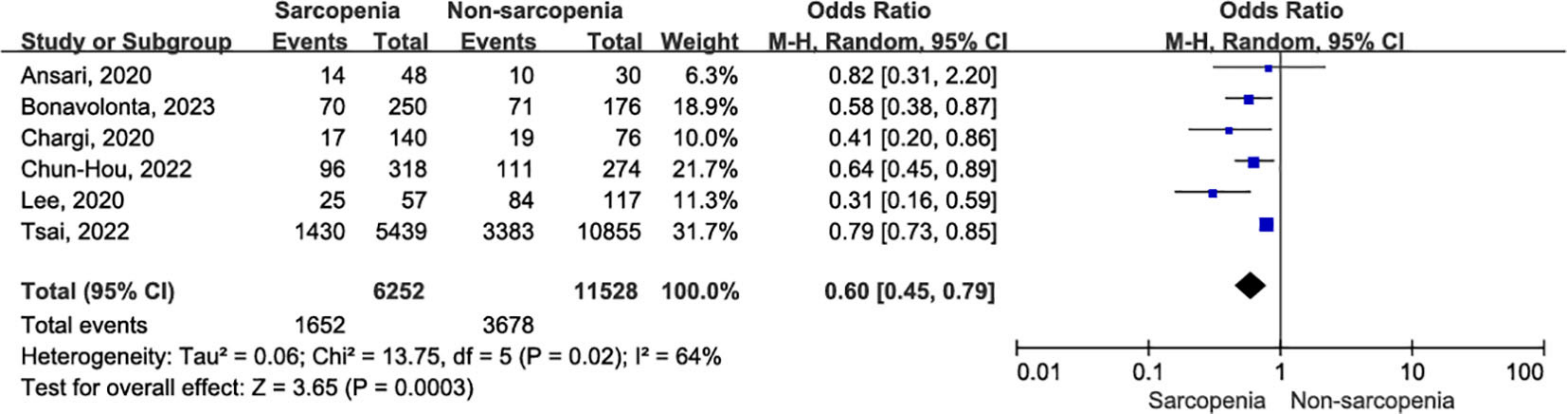
The forest plot of five-year OS.

Six original records reported five-year OS outcomes postoperatively in a cohort of 17,780 patients with OSCC (10, 32–34, 36, 37). The five-year OS was significantly lower in patients with sarcopenia compared to those without sarcopenia (**Figure 6**; OR = 0.60, 95% CI = 0.45-0.79, P = 0.0003). A further leave-one-out approach was performed to identify the possible sources of heterogeneity between studies due to significant heterogeneity (I^2^ = 64%, P = 0.02). Remarkably, even after excluding the study by Tsai et al. (10), which utilized bioelectrical impedance analysis (BIA) to define sarcopenia and evaluate its impact on postoperative survival in OSCC patients, sarcopenia remained significantly associated with poorer five-year OS (OR = 0.54, 95% CI = 0.41-0.70, P < 0.00001), with markedly reduced heterogeneity (I^2^ = 19%, P = 0.29). Online **Supplementary Data Sheet 3** (**Supplementary Table 4**) presents the sensitivity analysis for five-year OS.

#### 30-day mortality

Among the studies reviewed, only one investigation specifically addressed 30-day mortality, and a meta-analysis was not conducted. Notably, Shuang et al. (39) observed that patients with sarcopenia faced an elevated risk of mortality within 30 days following surgery (OR = 1.24, 95% CI = 1.08-1.52, P = 0.0052). Furthermore, adjusted multifactor logistic regression analysis corroborated this finding, demonstrating a significantly higher 30-day mortality rate in the sarcopenia group (OR = 1.12, 95% CI = 1.07-1.56, P = 0.0016).

#### Postoperative pneumonia

**Figure 7:**
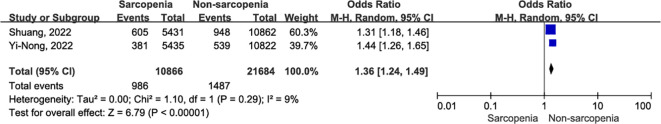
The forest plot of postoperative pneumonia.

In two original studies (35, 39), a total of 32,550 patients with OSCC reported the occurrence of pneumonia within 30 days after surgery. Patients with sarcopenia exhibited a higher risk of 30-day postoperative pneumonia compared to those without sarcopenia (**Figure 7**; OR = 1.36, 95% CI = 1.24-1.49, P < 0.00001). There was slight heterogeneity between articles (I^2^ = 9%, P = 0.29).

#### Surgical site infection

**Figure 8:**
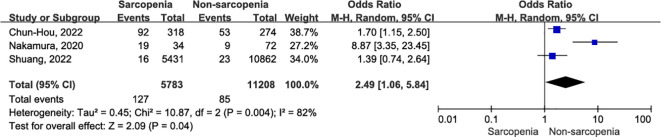
The forest plot of surgical site infection.

Surgical site infection 30 days after surgery were meticulously documented in three articles (36, 38, 39) involving a total of 16,991 patients with OSCC. The sarcopenia group showed a higher infection rate within the same 30-day postoperative period (**Figure 8**; OR = 2.49, 95% CI = 1.06-5.84, P = 0.04). However, there was significant heterogeneity among the included studies (I^2^ = 82%, P = 0.004). After excluding the study conducted by Nakamura et al. (38), patients with sarcopenia still exhibited an increased risk of surgical site infection 30 days after OSCC surgery compared to nonsarcopenic patients (OR = 1.61, 95% CI = 1.16-2.24, P = 0.005). Notably, there was a high degree of consistency among the included studies, and no evidence suggested the presence of heterogeneity (I^2^ = 0%, P = 0.60). A detailed interpretation of this literature suggests that the small sample size may be a potential source of heterogeneity. Additionally, undergoing resection surgery and flap reconstruction, factors such as insufficient blood supply to the flap and expansion of the surgical scope contributed to an elevated risk of postoperative infection. Online **Supplementary Data Sheet 3** (**Supplementary Table 5**) presents the sensitivity analysis for surgical site infection.

## Discussion

OSCC is one of the major causes of cancer death worldwide with high morbidity and mortality (40). Despite advancements in surgical techniques, optimized chemoradiotherapy strategies, and gene therapy, OSCC patients continue to experience low outcomes and quality of life (41, 42). Prognostic studies often center on surgical resection, with limited exploration of the relationship between sarcopenia and postoperative prognosis in OSCC. As pioneers in this field, we conducted a comprehensive systematic review and meta-analysis, critically appraising included studies to evaluate the impact of sarcopenia on survival and short-term outcomes following OSCC surgery. Ten studies involving 50611 patients were included. The results revealed that sarcopenia was highly prevalent with a worse three-year OS in OSCC patients. There was mild heterogeneity among the included studies when a subgroup analysis was performed. In addition, we also observed that sarcopenia was negatively correlated with one-year OS, five-year OS, increased 30-day mortality, postoperative pneumonia and surgical site infection.

Ten original studies, meeting the inclusion and exclusion criteria, were published within the last three years. Among these, three articles utilized BIA, while the remaining studies employed CT-measured SMI. Our systematic review and meta-analysis showed that sarcopenia significantly reduced three-year postoperative survival in patients with OSCC, albeit with high heterogeneity. In addition, the definitions and cut-off values of sarcopenia vary among studies. For example, in Ansari et al. low SMM was defined as a LSMI below 43.2 cm^2^/m^2^, this cut-off value was determined in a separate cohort of head and neck cancer patients (6). In this study, muscle cross-sectional area at the level of C3 was converted to CSA at the level of L3 using a previously published formula, and the LSMI was calculated by correcting SMM at the level of L3 for squared height. Different cut-off values for sarcopenia diagnosis may lead to significant heterogeneity between studies. However, as yet, there is no universal consensus on assessment methods for sarcopenia cut-off value, and cut-off points depend on the measurement technique and on the availability of reference studies and populations. To address this, we adopted a leave-one-out approach, meticulously examining the full text to identify sources of heterogeneity. We found that the heterogeneity between studies primarily stemmed from differences in sample and assessment tools for sarcopenia, and heterogeneity was significantly reduced by subgroup analysis. Similarly, one-year and five-year overall survival (OS) rates were significantly lower in patients with sarcopenia compared to those without, and this heterogeneity was also resolved through subgroup analysis based on sarcopenia assessment methods.

Furthermore, only one original study reported 30-day mortality after surgery, and we conducted a systematic review in which univariate and multifactorial logistic regression suggested a negative impact of sarcopenia—a finding consistent with other studies (25). Among three studies that reported surgical site infection 30 days after surgery, our meta-analysis showed that patients with sarcopenia were more likely to suffer surgical site infection. Notably, after excluding one of the studies that underwent flap reconstruction following prolonged resection, the heterogeneity was significantly reduced. The author posited that sarcopenia significantly increased the risk of postoperative surgical site infection. Even without assessing the flap donor site and remote areas, the risk of surgical site infection after free or pedicled flap reconstruction remained significantly higher than that following nonflap reconstruction. In fact, the surgical site infection rate in this study exceeded that observed in other studies, highlighting the influence of surgical type and prolonged exposure as variables contributing to heterogeneity. This conclusion aligns with the fact that OSCC patients faced a higher risk of postoperative surgical site infection compared to other clean-contaminated surgeries, particularly those who underwent resection surgery and free or pedicled flap reconstruction after extended resection (43–45).

The QUIPS and GRADE tools were used to assess the risk of bias and quality of evidence for the included studies, respectively. Due to differences in diagnostic tools for sarcopenia, there was significant heterogeneity between studies, and only one study reported 30-day postoperative mortality, we considered that the quality of evidence for three-year OS, one-year OS and 30-day mortality was moderate, and the quality of evidence for five-year OS was low. The incidence of preoperative COPD and dysphagia in the sarcopenia group was significantly higher than that in the non-sarcopenia group. The higher postoperative pneumonia risk in patients with sarcopenic OSCC compared with those with nonsarcopenic could be the result of various factors. Therefore, the quality of evidence for postoperative pneumonia was judged to be low. In addition, there were significant differences in the type and duration of surgery. In one of the included studies, resection surgery and flap reconstruction were performed, factors such as insufficient blood supply to the flap and expansion of the surgical scope contributed to an elevated risk of postoperative infection, the quality of evidence for surgical site infection was rated as very low based on multiple confounders. The results of subgroup analysis and sensitivity analysis demonstrated that the methodological quality of the studies was reliable, which increased the representativeness and generalizability of our conclusions.

An efficient and straightforward assessment tool for identifying high-risk surgical patients would be invaluable for medical decision-making. Sarcopenia, a critical component of frailty syndrome, is recognized as a risk factor for poor outcomes across various diseases, significantly increasing morbidity and mortality rates while being associated with adverse outcomes (46–48). Although no proper pharmacological interventions exist, many strategies have been proposed to manage sarcopenia, with nonpharmacological treatments proving most appropriate and effective. Accumulated evidence suggests that exercise and nutritional interventions partially reverse sarcopenia. For instance, resistance training, balance and aerobic exercises, and protein supplements have positively impacted muscle mass, strength, and physical performance (49–51). Consequently, it is reasonable to assume that timely and proper interventions for outpatient sarcopenic patients could prevent or delay disease onset, alleviating the heavy burden on both patients and the entire healthcare system.

Traditionally, MRI and CT scan have been considered the gold standards for noninvasive assessment of muscle quantity. The cutoff value for sarcopenia diagnosis is typically defined as an appendicular ASM or SMM that falls 2-2.5 standard deviations below the mean of a healthy young cohort. Remarkably, this criterion applies equally to both European and Asian populations (52, 53). However, high equipment costs, inconvenient portability, and the need for professional and trained personnel to perform the measurements have limited the widespread use of this equipment (13). To address these challenges, BIA prediction models have emerged as a promising alternative. BIA measures whole-body electrical conductivity and correlates well with other device-based measurements. Notably, BIA is noninvasive, time-saving, cost-effective, and radiation-free, making it suitable for extended screening and evaluation of sarcopenia in community primary care settings (54). In the context of oral squamous cell carcinoma (OSCC) patients, routine L3-CT scans are not commonly performed. However, we can easily obtain C3 level muscle mass during preoperative examinations and convert it to L3 using the equation described by Swartz et al. (55). Recognizing that assessment tools in the clinical environment may not always yield consistent results, by incorporating various diagnostic criteria and assessment tools for sarcopenia, our study aims to align with clinical practice and guide optimal perioperative management. We propose that potential differences should be addressed through data integration and meta-analysis to obtain robust conclusions.

Sarcopenia, a multifactorial condition, arises from a combination of several factors, including muscle motor unit loss, endocrine dyscrasia, malnutrition, systemic inflammatory response, and metabolic disorders (56). The decreased muscle mass and muscle strength in patients with sarcopenia deprived them of the opportunity for early postoperative ambulation. Simultaneously, malnutrition and weakness elevate the risk of postoperative respiratory failure, sepsis, and multiple organ dysfunction. These negative effects contribute to an increased incidence of postoperative complications and, in severe cases, premature mortality. Cachexia often coexists with these risk factors and manifests as severe muscle wasting, and sarcopenia is commonly observed in cachexia groups. Substantial evidence links sarcopenia to negative outcomes in malignant tumors (23, 24, 57). To enhance patient outcomes, preoperative interventions—such as exercise, nutritional support, and targeted medication—can have a positive impact, akin to other practices in elective surgery. However, the aggressive nature of malignant tumors necessitates timely surgical resection. In this context, early detection of sarcopenia becomes pivotal, providing crucial predictive information for optimizing outcomes. By incorporating exercise regimens and tailored nutritional support, we not only advocate for the inclusion of sarcopenia in early prognostic assessments for patients with OSCC but also emphasize its role in guiding personalized nutritional and optimal care goals. Consequently, early screening and multidisciplinary treatment emerge as essential strategies to mitigate adverse outcomes associated with sarcopenia.

The limitations of this systematic review and meta-analysis must be considered before reviewing the conclusions. First, meta-analyses inherently inherit the limitations of the studies they include. In our case, ten original records were retrospective cohort studies. While valuable, this study design has an inherent flaw: it tends to focus primarily on muscle mass parameters, often underestimating the significance of muscle strength and physical function assessment. Consequently, the predictive value of sarcopenia may be overstated. Second, the management of perioperative risk factors and effective interventions for complications may inadvertently lead to underestimating the negative impact of sarcopenia in clinical practice. In fact, the overestimation of sarcopenia prevalence and the potential for perioperative interventions further validate the association between sarcopenia and survival outcomes after OSCC surgery. Finally, the definition of sarcopenia varies across studies, introducing complexity. Factors such as race, sex, and age influence the cutoff value for diagnosing sarcopenia. Additionally, the overlap between sarcopenia and cancer cachexia complicates matters. As a result, the predictive value of sarcopenia might be inadvertently exaggerated. While our study convincingly demonstrates that sarcopenia predicts poor postoperative outcomes, further research is warranted. Investigating appropriate protocols to harmonize the diverse definitions of sarcopenia will enhance our findings and guide clinical practice effectively.

## Conclusion

Our systematic review and meta-analysis revealed that sarcopenia was associated with reduced three-year OS, one-year OS and five-year OS in OSCC patients postcurative resection. Additionally, 30-day mortality, postoperative pneumonia and surgical site infection in OSCC patients with sarcopenia were significantly higher than those in nonsarcopenic patients. Furthermore, more authoritative research should look to standardize the definition of sarcopenia and reconcile the limitations of this study to confirm and update our findings.

## Data Availability

The original contributions presented in the study are included in the article/**Supplementary Material**. Further inquiries can be directed to the corresponding author.
