# Supplementary material for: Association between sarcopenia and outcomes of surgically treated oral squamous cell carcinoma: a systematic review and meta‐analysis
Source: Front Oncol. 2024 Nov 1;14:1445956. doi: 10.3389/fonc.2024.1445956 (PMC11564163; doi:10.3389/fonc.2024.1445956)
Supplement: Supplementary file 2 [file DataSheet2.pdf]

Table 1

Characteristics of the included studies

Author, year	Design	Number of patients	Age	Sex, male, %	Group	Sample	Operation
Chun-Hou, 2022	Retrospective	N=592	56.8 (12)<br>51.3 (8)	518 (87.5)	Sarcopenia<br>Nonsarcopenia	n=318<br>n=274	OSCC curative surgery
Tsai, 2022	Retrospective	N=16294	55.0（15.0)<br>55.0（15.0)	14678 (90.1)	Sarcopenia<br>Nonsarcopenia	n=5439<br>n=10855	OSCC tumor resection and neck dissection
Chun-Hou, 2021	Retrospective	N=175	54.0 (9.5)<br>54.0 (10.4)	151 (86.3)	Sarcopenia<br>Nonsarcopenia	n=112<br>n=63	OSCC tumor resection+CRT
Shuang, 2022	Retrospective	N=16293	56.4 (11.1)<br>56.2 (11.3)	14632 (89.8)	Sarcopenia<br>Nonsarcopenia	n=5431<br>n=10862	OSCC tumor resection and neck dissection
Ansari, 2020	Retrospective	N=78	63.3 (10.9)<br>60.9 (8.8)	54 (69.2)	Sarcopenia<br>Nonsarcopenia	n=48<br>n=30	OSCC tumor resection+FFF
Bonavolonta, 2023	Retrospective	N=426	NA<br>NA	239 (56)	Sarcopenia<br>Nonsarcopenia	n=250<br>n=176	OSCC curative surgery
Chargi, 2020	Retrospective	N=216	63.6 (9.6)<br>60.3 (9.7)	143 (66)	Sarcopenia<br>Nonsarcopenia	n=140<br>n=76	OSCC curative surgery
Nakamura, 2020	Retrospective	N=106	65.5 (21)<br>68 (15.7)	66 (62.3)	Sarcopenia<br>Nonsarcopenia	n=34<br>n=72	OSCC curative surgery
Lee, 2020	Retrospective	N=174	53 (14)<br>50 (13)	159 (91.4)	Sarcopenia<br>Nonsarcopenia	n=57<br>n=117	OSCC curative surgery and radiotherapy
Yi-Nong, 2022	Retrospective	N=16257	NA<br>NA	NA	Sarcopenia<br>Nonsarcopenia	n=5435<br>n=10822	OSCC tumor resection and neck dissection

OSCC, oral cavity squamous cell carcinoma; CRT, chemoradiotherapy; FFF, fibula free flap.

Table 2

Diagnostic criteria for sarcopenia and outcomes of included studies

**Author, year**	**Diagnostic criteria**	**Outcomes**
Chun-Hou, 2022	SMA at the level of C3 was converted to SMA at the level of L3 (Swartz equation, SMI below 46.7 cm^2/m^2 for men and 30.3 cm^2/m^2 for women)	One-year, three-year and five-year OS and 30-day infection
Tsai, 2022	Bioelectrical Impedance Analysis (sarcopenia was defined as SMI of 2 SDs or more below the normal sex-specific means for young persons)	One-year, three-year and five-year OS
Chun-Hou, 2021	SMA at the level of C3 was converted to SMA at the level of L3 (Swartz equation, SMI below 46.7 cm^2/m^2 for men and 30.3 cm^2/m^2 for women)	One-year and three-year OS
Shuang, 2022	Bioelectrical Impedance Analysis (sarcopenia was defined as SMI of 2 SDs or more below the normal sex-specific means for young persons)	One-year, three-year OS, 30-day mortality, infection and pneumonia
Ansari, 2020	SMA at the level of C3 was converted to SMA at the level of L3 (Swartz equation, LSMI below 43.2 cm^2/m^2	One-year, three-year and five-year OS
Bonavolonta, 2023	SMA at the level of C3 was converted to SMA at the level of L3 (Swartz equation, SMI below 43.0 cm^2/m^2 for men and 41.0 cm^2/m^2 for women)	One-year, three-year and five-year OS
Chargi, 2020	SMA at the level of C3 was converted to SMA at the level of L3 (Swartz equation, LSMI below 43.0 cm^2/m^2	One-year, three-year and five-year OS
Nakamura, 2020	SMI at the level of L3 (SMI below 36.2 cm2/m2 for men and 31.0 cm2/m2 for women)	30-day infection
Lee, 2020	SMA at the level of C3 was converted to SMA at the level of L3 (Swartz equation, SMI below 52.4 cm^2/m^2 for men and 36.2 cm^2/m^2 for women)	One-year, three-year and five-year OS
Yi-Nong, 2022	Bioelectrical Impedance Analysis (sarcopenia was defined as SMI of 2 SDs or more below the normal sex-specific means for young persons)	30-day pneumonia

SMA, skeletal muscle area; C3, third cervical vertebra; L3, third lumbar vertebra; SMI, skeletal muscle index; SD, standard deviation; OS, overall survival.
