# Supplementary material for: Association between sarcopenia and outcomes of surgically treated oral squamous cell carcinoma: a systematic review and meta‐analysis
Source: Front Oncol. 2024 Nov 1;14:1445956. doi: 10.3389/fonc.2024.1445956 (PMC11564163; doi:10.3389/fonc.2024.1445956)
Supplement: Supplementary file 3 [file DataSheet3.pdf]

Risk of bias assessed by the Quality of Prognostic Studies (QUIPS).

study	study participation	study attrition	prognostic factor measurement	confounding measurement	outcome measurement	analysis and reporting
Chun-Hou, 2022	☆	★	☆	★★★	☆	☆
Tsai, 2022	☆	★	☆	★★★	☆	☆
Chun-Hou, 2021	☆	★	☆	★	☆	☆
Shuang, 2022	☆	★	☆	★	★	☆
Ansari, 2020	☆	★★★	☆	★	☆	☆
Bonavolonta, 2023	☆	★	☆	★★★	☆	☆
Chargi, 2020	☆	★	☆	★★★	☆	☆
Nakamura, 2020	☆	★	★	★★★	☆	★
Lee, 2020	☆	★	☆	★	☆	☆
Yi-Nong, 2022	☆	☆	☆	★★★	☆	☆

★★★, high; ★, moderate; ☆, low
