# Supplementary material for: Association between sarcopenia and outcomes of surgically treated oral squamous cell carcinoma: a systematic review and meta‐analysis
Source: Front Oncol. 2024 Nov 1;14:1445956. doi: 10.3389/fonc.2024.1445956 (PMC11564163; doi:10.3389/fonc.2024.1445956)
Supplement: Supplementary file 4 [file DataSheet4.pdf]

Table 3.

Sensitivity analysis of five-year OS.

Study	Statistics postremoval study
Ansari, 2020	OR = 0.58, 95% CI = 0.43-0.78, P = 0.0004; I^2 = 71%, P = 0.008.
Bonavolonta, 2023	OR = 0.59, 95% CI = 0.43-0.83, P = 0.002; I^2 = 66%, P = 0.02.
Chargi, 2020	OR = 0.63, 95% CI = 0.47-0.83, P = 0.001; I^2 = 64%, P = 0.03.
Chun-Hou, 2022	OR = 0.57, 95% CI = 0.39-0.83, P = 0.003; I^2 = 68%, P = 0.01.
Lee, 2020	OR = 0.69, 95% CI = 0.56-0.84, P = 0.0002; I^2 = 37%, P = 0.17.
Tsai, 2020	OR = 0.54, 95% CI = 0.41-0.70, P < 0.00001; I^2 = 19%, P = 0.29.

Table 4.

Sensitivity analysis of surgical site infection.

Study	Statistics postremoval study
Chun-Hou, 2022	OR = 3.38, 95% CI = 0.55-20.73, P = 0.19; I^2 = 90%, P = 0.002.
Nakamura, 2020	OR = 1.61, 95% CI = 1.16-2.24, P = 0.005; I^2 = 0%, P = 0.60.
Shuang, 2022	OR = 3.64, 95% CI = 0.72-18.33, P = 0.12; I^2 = 90%, P = 0.002.
