# Supplementary material for: Association between sarcopenia and outcomes of surgically treated oral squamous cell carcinoma: a systematic review and meta‐analysis
Source: Front Oncol. 2024 Nov 1;14:1445956. doi: 10.3389/fonc.2024.1445956 (PMC11564163; doi:10.3389/fonc.2024.1445956)
Supplement: Supplementary file 5 [file Table1.docx]

**Appendix**

Detailed search strategies from PubMed, Embase, Cochrane Library, Medline and Web of Science. The searches will be rerun prior to the final analyses and any further studies identified will be included.

| **Search terms for PubMed** | |
| --- | --- |
| **Search** | Query |
| **#1** | "Sarcopenia"[MeSH Terms] |
| **#2** | "Sarcopenia"[Title/Abstract] OR "Sarcopenias"[Title/Abstract] OR "Sarcopenic"[Title/Abstract] OR "Muscle mass"[Title/Abstract] OR "Muscle area"[Title/Abstract] |
| **#3** | **#1** OR **#2** |
| **#4** | "Oral"[MeSH Terms] |
| **#5** | "Oral"[Title/Abstract] OR "Mouth"[Title/Abstract] OR "Oral Cavity"[Title/Abstract] OR "Cavity, Oral"[Title/Abstract] OR "Cavitas Oris"[Title/Abstract] OR "Vestibule of the Mouth"[Title/Abstract] OR "Vestibule Oris"[Title/Abstract] OR "Oral Cavity Proper"[Title/Abstract] OR "Mouth Cavity Proper"[Title/Abstract] OR "Cavitas oris propria"[Title/Abstract] |
| **#6** | **#4** OR **#5** |
| **#7** | "Carcinoma"[MeSH Terms] |
| **#8** | "Carcinoma"[Title/Abstract] OR "Tumor"[Title/Abstract] OR "Neoplasm"[Title/Abstract] OR "Tumors"[Title/Abstract] OR "Neoplasia"[Title/Abstract] OR "Neoplasias"[Title/Abstract] OR "Cancer"[Title/Abstract] OR "Cancers"[Title/Abstract] OR "Malignant Neoplasm"[Title/Abstract] OR "Malignancy"[Title/Abstract] OR "Malignancies"[Title/Abstract] OR "Malignant Neoplasms"[Title/Abstract] OR "Neoplasm, Malignant"[Title/Abstract] OR "Neoplasms, Malignant"[Title/Abstract] OR "Benign Neoplasms"[Title/Abstract] OR "Neoplasm, Malignant"[Title/Abstract] OR "Benign Neoplasm"[Title/Abstract] OR "Neoplasms, Benign"[Title/Abstract] OR "Neoplasm, Benign"[Title/Abstract] |
| **#9** | **#7** OR **#8** |
| **#10** | **#6** AND **#9** |
| **#11** | **#3** AND **#10** |

| **Search terms for EMBASE** | |
| --- | --- |
| **Search** | Query |
| **#1** | ‘Sarcopenia’/exp |
| **#2** | ‘Sarcopenia’: ab, ti |
| **#3** | ‘Sarcopenias’: ab, ti |
| **#4** | ‘Sarcopenic’: ab, ti |
| **#5** | ‘Muscle mass’/exp |
| **#6** | ‘Muscle mass’: ab, ti. |
| **#7** | ‘Muscle area’/exp |
| **#8** | ‘Muscle area’: ab, ti. |
| **#9** | ‘Muscle areas’: ab, ti. |
| **#10** | **#1** OR **#2** OR **#3** OR **#4** OR **#5** OR **#6** OR **#7** OR **#8** OR **#10** |
| **#11** | ‘Oral’/exp |
| **#12** | ‘Oral’: ab, ti. |
| **#13** | ‘Mouth’: ab, ti. |
| **#14** | ‘Oral Cavity’: ab, ti. |
| **#15** | ‘Cavity, Oral’: ab, ti. |
| **#16** | ‘Cavitas Oris’: ab, ti. |
| **#17** | ‘Vestibule of the Mouth’: ab, ti. |
| **#18** | ‘Vestibule Oris’: ab, ti. |
| **#19** | ‘Oral Cavity Proper’: ab, ti. |
| **#20** | ‘Mouth Cavity Proper’: ab, ti. |
| **#21** | ‘Cavitas oris propria’: ab, ti. |
| **#22** | **#11** OR **#12** OR **#13** OR **#14** OR **#15** OR **#16** OR **#17** OR **#18** OR **#19** OR **#20** OR **#21** |
| **#23** | ‘Carcinoma’/exp |
| **#24** | ‘Carcinoma’: ab, ti. |
| **#25** | ‘Tumor’: ab, ti. |
| **#26** | ‘Neoplasm’: ab, ti. |
| **#27** | ‘Tumors’: ab, ti. |
| **#28** | ‘Neoplasia’: ab, ti. |
| **#29** | ‘Neoplasias’: ab, ti. |
| **#30** | ‘Cancer’: ab, ti. |
| **#31** | ‘Cancers’: ab, ti. |
| **#32** | ‘Malignant Neoplasm’: ab, ti. |
| **#33** | ‘Malignancy’: ab, ti. |
| **#34** | ‘Malignancies’: ab, ti. |
| **#35** | ‘Malignant Neoplasms’: ab, ti. |
| **#36** | ‘Neoplasm, Malignant’: ab, ti. |
| **#37** | ‘Neoplasms, Malignant’: ab, ti. |
| **#38** | ‘Benign Neoplasms’: ab, ti. |
| **#39** | ‘Benign Neoplasm’: ab, ti. |
| **#40** | ‘Neoplasms, Benign’: ab, ti. |
| **#41** | ‘Neoplasm, Benign’: ab, ti. |
| **#42** | **#23** OR **#24** OR **#25** OR **#26** OR **#27** OR **#28** OR **#29** OR **#30** OR **#31** OR **#32** OR **#33** OR **#34** OR **#35** OR **#36** OR **#37** OR **#38** OR **#39** OR **#40** OR **#41** |
| **#43** | **#22** AND **#42** |
| **#44** | **#10** AND **#43** |

| **Search terms for the Cochrane Library (in Title, Abstract, Keyword)** | |
| --- | --- |
| **Search** | Query |
| **#1** | MeSH descriptor: [Sarcopenia] explode all trees |
| **#2** | Sarcopenia |
| **#3** | Sarcopenias |
| **#4** | sarcopenic |
| **#5** | MeSH descriptor: [Muscle mass] explode all trees |
| **#6** | Muscle mass |
| **#7** | Skeletal muscle mass |
| **#8** | MeSH descriptor: [Muscle area] explode all trees |
| **#9** | Muscle area |
| **#10** | Muscle areas |
| **#11** | **#1** OR **#2** OR **#3** OR **#4** OR **#5** OR **#6** OR **#7** OR **#8** OR **#9** OR **#10** |
| **#12** | MeSH descriptor: [Oral] explode all trees |
| **#13** | Oral |
| **#14** | Mouth |
| **#15** | Oral Cavity |
| **#16** | Cavity, Oral |
| **#17** | Cavitas Oris |
| **#18** | Vestibule of the Mouth |
| **#19** | Vestibule Oris |
| **#20** | Oral Cavity Proper |
| **#21** | Mouth Cavity Proper |
| **#22** | Cavitas oris propria |
| **#23** | **#12** OR **#13** OR **#14** OR **#15** OR **#16** OR **#17** OR **#18** OR **#19** OR **#20** OR **#21 #22** |
| **#24** | MeSH descriptor: [Carcinoma] explode all trees |
| **#25** | Carcinoma |
| **#26** | Tumor |
| **#27** | Neoplasm |
| **#28** | Tumors |
| **#29** | Neoplasia |
| **#30** | Neoplasias |
| **#31** | Cancer |
| **#32** | Cancers |
| **#33** | Malignant Neoplasm |
| **#34** | Malignancy |
| **#35** | Malignancies |
| **#36** | Malignant Neoplasms |
| **#37** | Neoplasm, Malignant |
| **#38** | Neoplasms, Malignant |
| **#39** | Benign Neoplasms |
| **#40** | Benign Neoplasm |
| **#41** | Neoplasms, Benign |
| **#42** | Neoplasm, Benign |
| **#43** | **#24** OR **#25** OR **#26** OR **#27** OR **#28** OR **#29** OR **#30** OR **#31** OR **#32** OR **#33** OR **#34** OR **#35** OR **#36** OR **#37** OR **#38** OR **#39** OR **#40** OR **#41** OR **#42** |
| **#44** | **#23** AND **#43** |
| **#45** | **#11** AND **#44** |
| **Search terms for MEDLINE** | |
| **Search** | Query |
| **#1** | Sarcopenia. ab, ti. |
| **#2** | Sarcopenias. ab, ti. |
| **#3** | Sarcopenic. ab, ti. |
| **#4** | Muscle mass. ab, ti. |
| **#5** | Skeletal muscle mass. ab, ti. |
| **#6** | Muscle area. ab, ti. |
| **#7** | Muscle areas |
| **#8** | **#1** OR **#2** OR **#3** OR **#4** OR **#5** OR **#6** OR **#7** |
| **#9** | Oral. ab, ti. |
| **#10** | Mouth. ab, ti. |
| **#11** | Oral Cavity. ab, ti. |
| **#12** | Cavity, Oral. ab, ti. |
| **#13** | Cavitas Oris. ab, ti. |
| **#14** | Vestibule of the Mouth. ab, ti. |
| **#15** | Vestibule Oris. ab, ti. |
| **#16** | Oral Cavity Proper. ab, ti. |
| **#17** | Mouth Cavity Proper. ab, ti. |
| **#18** | Cavitas oris propria. ab, ti. |
| **#19** | **#9** OR **#10** OR **#11** OR **#12** OR **#13** OR **#14** OR **#15** OR **#16** OR **#17** OR **#18** |
| **#20** | Carcinoma. ab, ti. |
| **#21** | Tumor. ab, ti. |
| **#22** | Neoplasm. ab, ti. |
| **#23** | Tumors. ab, ti. |
| **#24** | Neoplasia. ab, ti. |
| **#25** | Neoplasias. ab, ti. |
| **#26** | Cancer. ab, ti. |
| **#27** | Cancers. ab, ti. |
| **#28** | Malignant Neoplasm. ab, ti. |
| **#29** | Malignancy. ab, ti. |
| **#30** | Malignancies. ab, ti. |
| **#31** | Malignant Neoplasms. ab, ti. |
| **#32** | Neoplasm, Malignant. ab, ti. |
| **#33** | Neoplasms, Malignant. ab, ti. |
| **#34** | Benign Neoplasms. ab, ti. |
| **#35** | Benign Neoplasm. ab, ti. |
| **#36** | Neoplasms, Benign. ab, ti. |
| **#37** | Neoplasm, Benign. ab, ti. |
| **#38** | **#20** OR **#21** OR **#22** OR **#23** OR **#24** OR **#25** OR **#26** OR **#27** OR **#28** OR **#29** OR **#30** OR **#31** OR **#32** OR **#33** OR **#34** OR **#35** OR **#36** OR **#37** |
| **#39** | **#19** AND **#38** |
| **#40** | **#8** AND **#39** |

| **Search terms for Web of Science** | |
| --- | --- |
| **Search** | Query |
| **#1** | Sarcopenia (Topic) OR Sarcopenia (Title) OR Sarcopenia (Abstract) OR Sarcopenias (Topic) OR Sarcopenias (Title) OR Sarcopenias (Abstract) OR sarcopenic (Topic) OR sarcopenic (Title) OR sarcopenic (Abstract) |
| **#2** | Muscle mass (Topic) OR Muscle mass (Title) OR Muscle mass (Abstract) OR Muscle area (Topic) OR Muscle area (Title) OR Muscle area (Abstract) OR Muscle areas (Topic) OR Muscle areas (Title) OR Muscle areas (Abstract) |
| **#3** | **#1** OR **#2** |
| **#4** | Oral (Topic) OR Oral (Title) OR Oral (Abstract) OR Mouth (Topic) OR Mouth (Title) OR Mouth (Abstract) OR Oral Cavity (Topic) OR Oral Cavity (Title) OR Oral Cavity (Abstract) OR Cavity, Oral (Topic) OR Cavity, Oral (Title) OR Cavity, Oral (Abstract) OR Cavitas Oris (Topic) OR Cavitas Oris (Title) OR Cavitas Oris (Abstract) OR Vestibule of the Mouth (Topic) OR Vestibule of the Mouth (Title) OR Vestibule of the Mouth (Abstract) OR Vestibule Oris (Topic) OR Vestibule Oris (Title) OR Vestibule Oris (Abstract) OR Oral Cavity Proper (Topic) OR Oral Cavity Proper (Title) OR Oral Cavity Proper (Abstract) OR Mouth Cavity Proper (Topic) OR Mouth Cavity Proper (Title) OR Mouth Cavity Proper (Abstract) OR Cavitas oris propria (Topic) OR Cavitas oris propria (Title) OR Cavitas oris propria (Abstract) |
| **#5** | Carcinoma (Topic) OR Carcinoma (Title) OR Carcinoma (Abstract) OR Tumor (Topic) OR Tumor (Title) OR Tumor (Abstract) OR Neoplasm (Topic) OR Neoplasm (Title) OR Neoplasm (Abstract) OR Tumors (Topic) OR Tumors (Title) OR Tumors (Abstract) OR Neoplasia (Topic) OR Neoplasia (Title) OR Neoplasia (Abstract) OR Neoplasias (Topic) OR Neoplasias (Title) OR Neoplasias (Abstract) OR Cancer (Topic) OR Cancer (Title) OR Cancer (Abstract) OR Cancers (Topic) OR Cancers (Title) OR Cancers (Abstract) OR Malignant Neoplasm (Topic) OR Malignant Neoplasm (Title) OR Malignant Neoplasm (Abstract) OR Malignancy (Topic) OR Malignancy (Title) OR Malignancy (Abstract) OR Malignancies (Topic) OR Malignancies (Title) OR Malignancies (Abstract) OR Malignant Neoplasms (Topic) OR Malignant Neoplasms (Title) OR Malignant Neoplasms (Abstract) OR Neoplasm, Malignant (Topic) OR Neoplasm, Malignant (Title) OR Neoplasm, Malignant (Abstract) OR Neoplasms, Malignant (Topic) OR Neoplasms, Malignant (Title) OR Neoplasms, Malignant (Abstract) OR Benign Neoplasms (Topic) OR Benign Neoplasms (Title) OR Benign Neoplasms (Abstract) OR Benign Neoplasm (Topic) OR Benign Neoplasm (Title) OR Benign Neoplasm (Abstract) OR Neoplasms, Benign (Topic) OR Neoplasms, Benign (Title) OR Neoplasms, Benign (Abstract) OR Neoplasm, Benign (Topic) OR Neoplasm, Benign (Title) OR Neoplasm, Benign (Abstract) |
| **#6** | **#4** AND **#5** |
| **#7** | **#3** AND **#6** |
